# Supplementary material for: LongTR: genome-wide profiling of genetic variation at tandem repeats from long reads
Source: Genome Biol. 2024 Jul 4;25:176. doi: 10.1186/s13059-024-03319-2 (PMC11229021; doi:10.1186/s13059-024-03319-2)
Supplement: Supplementary file 1 — Additional file 1. Contains supplementary figures S1-S7, tables S1-S2 and their legends. [file 13059_2024_3319_MOESM1_ESM.pdf]

Supplementary Figures

Fig S1

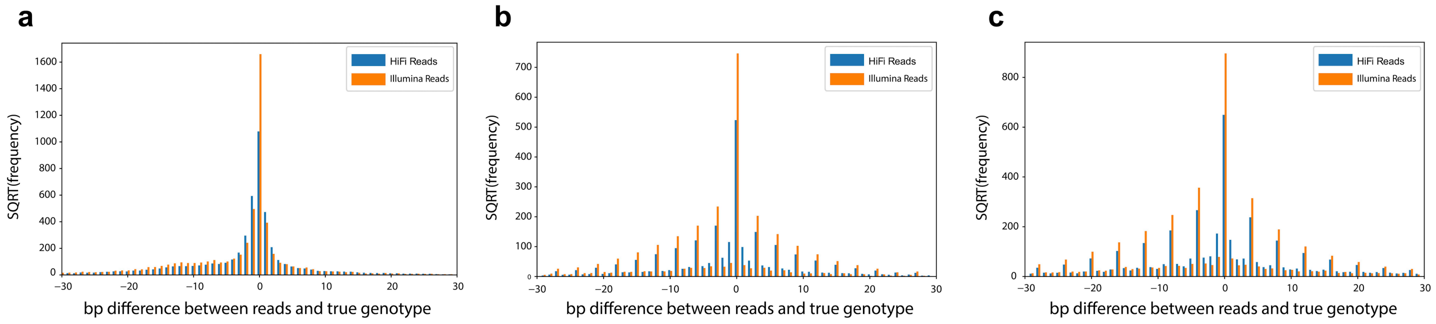

Base pair error distribution for PacBio HiFi and Illumina reads. Bars show the square root of the frequency of error sizes for PacBio HiFi (blue) and Illumina (orange) reads from sample HG002. **(a)** bp difference with the maximum likelihood genotype for homopolymers with total length between 20-60bp; **(b)** bp difference with the maximum likelihood genotype for trinucleotides with total length between 30-100bp; **(c)** bp difference with the maximum likelihood genotype for tetranucleotides with total length between 40-100bp. Notably, for **b-c**, PacBio reads show a higher rate of errors that are not a multiple of the repeat unit length.

Fig S2

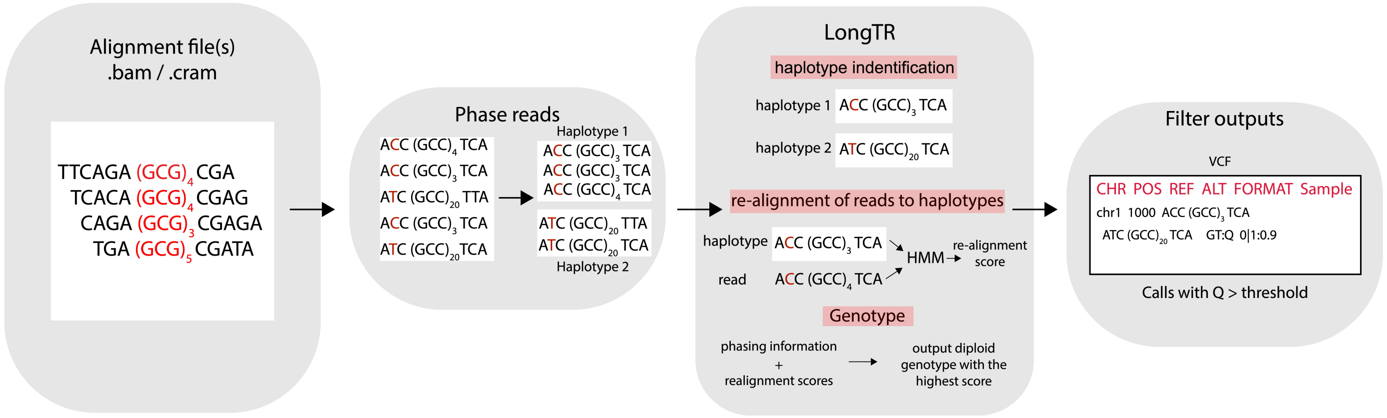

LongTR workflow. Users may first optionally haplotag (phase) the input reads. The aligned reads, along with a reference set of TRs, are input to LongTR for genotyping. LongTR uses a clustering strategy combined with partial order alignment to infer consensus haplotypes from error-prone reads, followed by sequence realignment using a Hidden Markov Model to infer the highest scoring genotypes. Finally, it outputs a VCF file with inferred genotypes, quality scores, and other fields that can be used to further filter low quality calls.

Fig S3

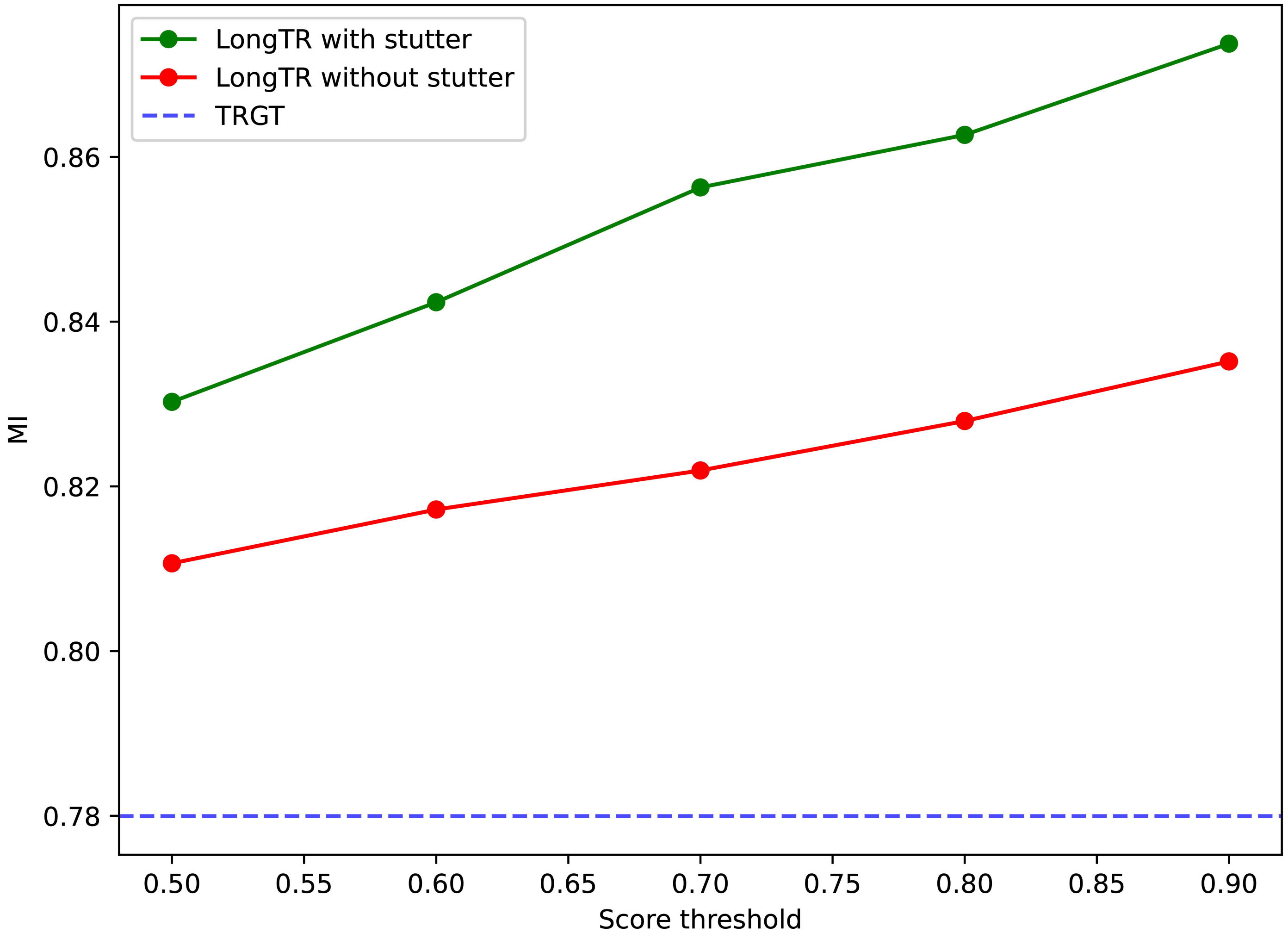

Mendelian Inheritance for an Ashkenazi Trio at homopolymer TRs as a function of LongTR quality score. The x-axis shows LongTR quality score threshold, and the y-axis shows the percent of genotyped loci that follow Mendelian Inheritance. The green line shows LongTR with stutter error modeling, the red line shows LongTR without using stutter error modeling, and the blue dashed line shows TRGT. Each locus was included if all 3 samples passed the score threshold. Loci where all three samples were homozygous for the reference allele were excluded from the analysis.

Fig S4

a

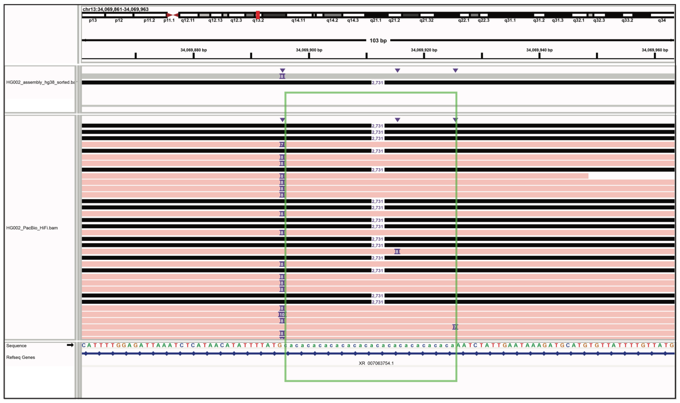

b

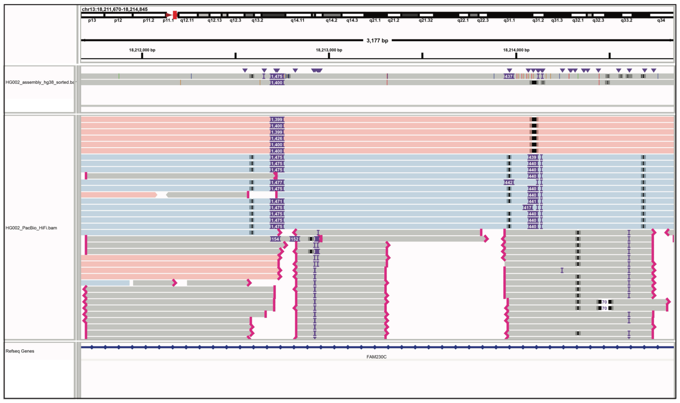

IGV screenshots of examples where LongTR matches the assembly but TRGT does not. The top window shows alignments of the maternal and paternal assemblies and the bottom window shows aligned HiFi reads to GRCh38. Red and blue denote PacBio HiFi reads from the two haplotypes of HG002 based on haplotag information. Gray reads have no haplotag information. The screenshot was generated using the Integrative Genomics Viewer. **(a)** LongTR detects structural deletions resulting in null TR alleles**.** The repeat boundary is denoted by the green box. Note no reads from the haplotype labeled in blue are visible since those reads harbor a 2,731bp deletion at this locus (denoted by the black lines). **(b)** The window shows the repeat boundary. TRGT reports deletions in regions with a high number of truncated reads.

Fig S5

a **b**

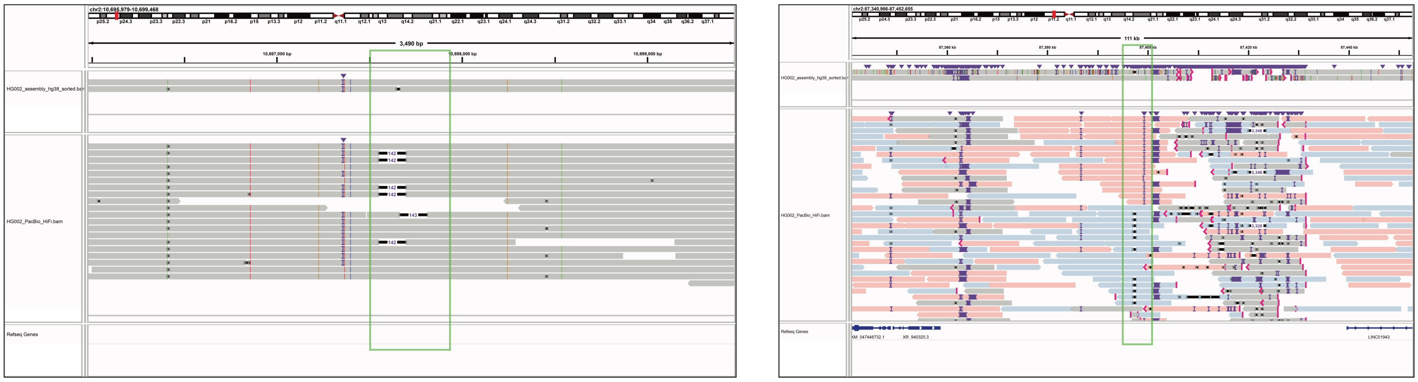

IGV screenshots comparing the HG002 assembly alleles with PacBio HiFi reads aligned to GRCh38 at loci where TRGT and LongTR agree but do not match the assembly. The top window shows alignments of the maternal and paternal assemblies and the bottom window shows aligned HiFi reads. Red and blue denote PacBio HiFi reads from the two haplotypes of HG002 based on haplotag information. Gray reads have no haplotag information. The repeat boundary is denoted by the green box. **(a)** shows a VNTR with a 141bp repeat unit and total length of 474bp on GRCh38. This is an example of a highly homozygous region where assembly alleles are most likely incorrect as neither contain the 142bp deletion supported by multiple reads. **(b)** shows a VNTR with repeat unit of CCAAGCCAG and total length of 5,180bp on GRCh38 where the insertion on the red haplotype is missing from the assembly due to a nearby highly divergent tandem repeat that causes a break in assembly alignment.

Fig S6

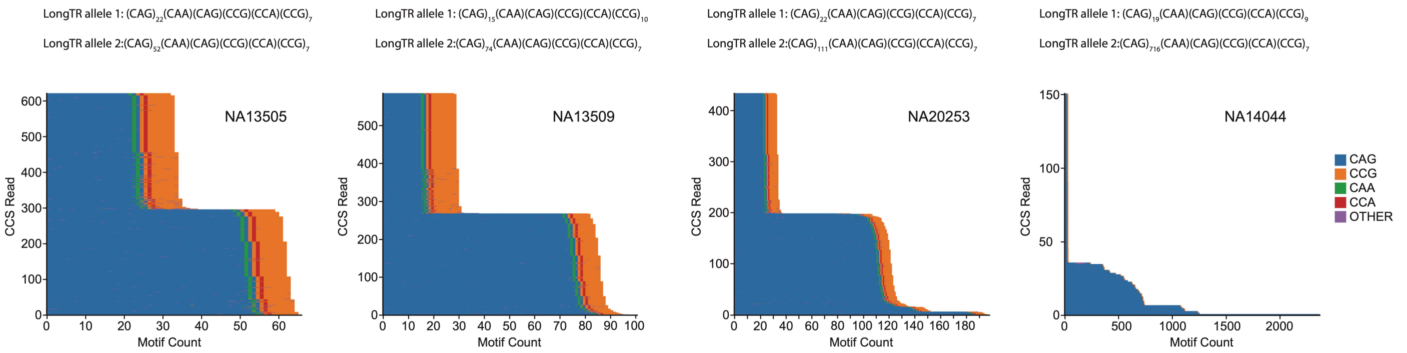

Waterfall plots for reads from samples with an *HTT* expansion genotyped by LongTR. Each row in each plot represents a single HiFi read. The x-axis shows the count of each motif identified in each read. LongTR precisely identified heterozygous allele sequences for which the repeat unit with the expansion can be inferred. From the left, plots show reads for samples NA13505, NA13509, NA20253, and NA14044. Plots were generated using TRviz.

Fig S7

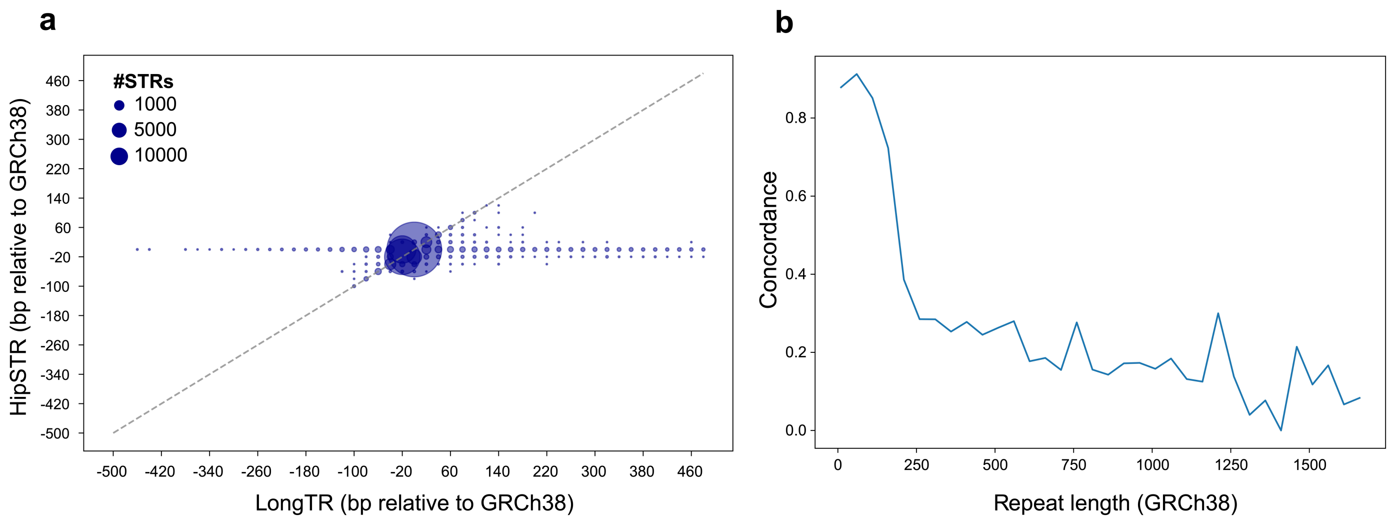

Comparison between STR calls by HipSTR using Illumina short reads vs. LongTR on PacBio HiFi reads from sample HG002. (a) Length concordance between HipSTR (short reads) vs. LongTR (long reads) genotypes. The x-axis and y-axis show the average base pair difference from the GRCh38 reference genome for the two alleles at each TR. Bubble size shows the number of points at each coordinate. **(b) Most discordances are at longer TRs.** The x-axis shows the repeat length on hg38. The y-axis shows the fraction of concordant calls between HipSTR and LongTR.

Supplementary Tables

Table S1

Inferred error model parameters at homopolymer TRs in PacBio HiFi reads from HG002. Parameters u and d indicate the probability to see an expansion or deletion error in each read. The step size of errors (length difference from the true allele in bp) is characterized by a geometric distribution with a parameter 'p'. We computed separate error models for different length ranges of homopolymers. As expected, errors become larger and more frequent with the increase in homopolymer repeat length.

Homopolymer repeat length (bp)	Step-size (p)	Deletion coefficient (d)	Insertion coefficient (u)
10-19	0.92	0.15	0.10
20-29	0.86	0.23	0.13
30-39	0.80	0.28	0.14
40-49	0.75	0.31	0.14
>=50	0.71	0.34	0.15

Table S2

Base pair differences from the reference genome at four TR loci were measured by LongTR using PacBio HiFi data. The first four patients have expansions in *HTT*. The next three have expansions in *FMR1*. HEK293 has no known expansion at these loci. None of the samples have expansions in *ATXN10* or *C9orf72*.

Sample	HTT		ATXN10		C9orf72		FMR1	
	TRGT	LongTR	TRGT	LongTR	TRGT	LongTR	TRGT	LongTR
**NA12505**	9,96	9,96	0,20	0,20	24,42	24,42	30,33	30,33
**NA13509**	-3,168	-3,168	-10,30	-10,30	12,18	12,18	30,33	30,33
**NA20253**	9,273	9,276	-5,10	-5,10	-6,-6	-6,-6	0,0	0,0
**NA14044**	6,1876	6,2115	-10,-10	-10,-10	-6,-6	-6,-6	30,33	30,33
**NA13664**	-6,0	-6,0	0,0	0,0	-6,-6	-6,-6	30,99	30,99
**NA06896**	-18,3	-18,3	-5,20	-5,20	-6,30	-6,30	9,509	9,426
**NA07537**	-18,-6	-18,-6	-10,10	-10,10	-6,-6	-6,-6	27,954	27,1000
**HEK293**	-6,-3	-6,-3	10,10	10,10	-6,6	-6,6	30,33	30,30
