# Supplementary material for: LongTR: genome-wide profiling of genetic variation at tandem repeats from long reads
Source: Genome Biol. 2024 Jul 4;25:176. doi: 10.1186/s13059-024-03319-2 (PMC11229021; doi:10.1186/s13059-024-03319-2)
Supplement: Supplementary file 2 — Additional file 2. Review history. [file 13059_2024_3319_MOESM2_ESM.docx]

**1^st^ round**

**Reviewer 1**

Jam et al report a new tool, LongTR, to genotype tandem repeat size from long-read (ONT or PacBio) sequence data, given a catalog of tandem repeats (TRs) of interest. To evaluate the performance of this tool, the authors (1) compare LongTR to another existing method, Tandem Repeat Genotyping Tool (TRGT), for genotyping a catalogue of 937,122 TRs in the HG002 sample; (2) genotype three samples from a trio family (HG002, HG003 and HG004) to assess Mendelian inheritance; (3) compare LongTR to adVNTR on genotyping repeats of >6bp unit length; (4) compare the genotypes from LongTR on PacBio Hifi data to that on ONT duplex data, and (5) compare HipSTR calls from Illumina data to LongTR calls from PacBio data. In general, the authors show that their tool outperforms, or works as well as, existing methods in most of the comparisons. This tool is built upon HipSTR, which is a well-founded method for TR genotyping. Their multi-sample haplotyping approach is novel and useful. The manuscript is also well-written.

Specific comments:

1. For ONT's long reads, the group benchmarked using recently released duplex reads. Have the authors tried its performance on simplex reads?

2. Can the authors comment on how the tool handles regions with multiple motifs?

3. The authors phase, and comment on phasing PacBio HiFi data - can they similarly comment on whether ONT genotyping accuracy is improved (or is expected to improve) after phasing?

4. Lines 11-12: "TRGT and LongTR showed 97.6% and 98.3% length concordance allowing for 1 bp differences.": This is slightly confusing - the output of repeat genotyping would presumably be in repeat counts, rather than repeat length in sequence base pairs. Does this mean that the outputs differ by 1bp of total length in base pairs, or that 1bp of difference is allowed in the motif? If the latter, this would likely affect the genotyping outcome by more than a few repeat counts.

5. "genotypes obtained from ONT duplex and PacBio HiFi (88% allowing for 1bp off; Fig. 3a) with the latter showing higher concordance with the assembly (99% for PacBio HiFi vs 87% for ONT, allowing for 1bp off).": Earlier in the manuscript when comparing PacBio HiFi reads to the assembly, the authors mentioned that "LongTR showed […] 98.3% length concordance allowing for 1 bp differences." Can the authors clarify the accuracy of their tool?

6. In "Phasing information", How did authors determine the 20% cutoff for unphased reads?

7. The figure legends for Fig 2.a. and Fig. 2.b seem to be swapped; 2a shows mendelian consistency, while 2b shows Concordance with genotype assembly.

8. Figure 3 needs to be replaced with higher resolution images.

**Reviewer 2**

Jam et al present LongTR, a method for genotyping short tandem repeats (STRs) and variable number tandem repeats (VNTRs) in long read data. The method is benchmarked against TRGT, a competing tool, and the performance of the method is demonstrated on both PacBio and ONT data. It is a timely work of interest to researchers in the field, and contains promising advances.

I would draw the authors' attention to:

1. A number of STR and VNTR tools are cited, but not included in benchmarking. It would be beneficial to include a comparative evaluation of LongTR against all cited tools, specially STRaglr, given its integration into ONT's Epi2Me pipelines. This should be completed for publication of any bioinformatics tool in a field with multiple existing methods.

2. It should be noted that LongTR is benchmarked against TRGT v0.4.0. This is not the version of TRGT that ultimately is the subject of the cited TRGT paper (v0.5.0). A repeat of the benchmarking of LongTR against TRGT v0.5.0 is needed prior to publication. TRGT's current version is 0.8.0; significantly newer than that which LongTR is compared to here.

3. Please include information about how the software peformance was tested - computing environment, CPUs, total and type of memory used, and whether software was run in isolation (if the computing environment is shared, such as a HPC where other jobs' IO requirements may impact benchmarking). This is needed for reproducibility and clarity.

Minor/tyopgraphical

1. The URL in the review copy abstract has an extra period at the end of the github URL, which causes it to fail.

2. I found the order of presentation of LongTR performance difficult to follow in places. I would suggest presenting this as a benchmark against ground truth sequence first, then against competing software, rather than the reverse.

3. Are the reads that can be resolved with longer ONT reads but not shorter PacBio reads in known hard-to-map regions for long read sequencing, such as those previously identified by the GIAB Consortium (e.g. https://www.cell.com/cell-genomics/pdfExtended/S2666-979X(22)00058-1)?

4. The release notes for TRGT v0.5.0 mention the incorporation of SNP information similar to the haplotag read phase information used by LongTR and TRiCoLOR. The statement on page 4 regarding the uniqueness of LongTR's approach among long read TR genotypers in using read phase information needs revision.

5. LongTR needs to be packaged into a release on Github and a version number assigned prior to publication. Please release the version of the software described in the manuscript as version 1.0 per standard semantic versioning for released software.

**Authors’ response**

Reviewer 1:

Jam et al report a new tool, LongTR, to genotype tandem repeat size from long-read (ONT or PacBio) sequence data, given a catalog of tandem repeats (TRs) of interest. To evaluate the performance of this tool, the authors (1) compare LongTR to another existing method, Tandem Repeat Genotyping Tool (TRGT), for genotyping a catalogue of 937,122 TRs in the HG002 sample; (2) genotype three samples from a trio family (HG002, HG003 and HG004) to assess Mendelian inheritance; (3) compare LongTR to adVNTR on genotyping repeats of >6bp unit length; (4) compare the genotypes from LongTR on PacBio Hifi data to that on ONT duplex data, and (5) compare HipSTR calls from Illumina data to LongTR calls from PacBio data. In general, the authors show that their tool outperforms, or works as well as, existing methods in most of the comparisons. This tool is built upon HipSTR, which is a well-founded method for TR genotyping. Their multi-sample haplotyping approach is novel and useful. The manuscript is also well-written.

Specific comments:

1. For ONT's long reads, the group benchmarked using recently released duplex reads. Have the authors tried its performance on simplex reads?

We ran LongTR on the recently released ONT simplex SUP (super accuracy) sequence data, which according to the Oxford Nanopore webpage has the highest quality among simplex sequencing data (Q23). We saw similar performance on 12,503 repeats on chromosome 21 between the duplex dataset and simplex SUP in terms of concordance with assembly, with duplex being slightly more concordant (88.9% vs 88.7%). We now report these results in the main text.

2. Can the authors comment on how the tool handles regions with multiple motifs?

LongTR doesn’t require motif information to genotype, and so it can handle multiple motifs as well as perfect repeats. We now clarify this in the Methods in “Overview of the LongTR method”.

3. The authors phase, and comment on phasing PacBio HiFi data - can they similarly comment on whether ONT genotyping accuracy is improved (or is expected to improve) after phasing?

We phased duplex read using Whatshap v2.2 with DeepVariant small variant calls as input, and we saw an improvement of 2% in assembly concordance, with 87% concordance with unphased data and 89% for phased data on 12,503 repeats on chromosome 21. We now report these results in the main text.

4. Lines 11-12: "TRGT and LongTR showed 97.6% and 98.3% length concordance allowing for 1 bp differences.": This is slightly confusing - the output of repeat genotyping would presumably be in repeat counts, rather than repeat length in sequence base pairs. Does this mean that the outputs differ by 1bp of total length in base pairs, or that 1bp of difference is allowed in the motif? If the latter, this would likely affect the genotyping outcome by more than a few repeat counts.

Both LongTR and TRGT report the inferred allele sequence at each repeat. The 1bp difference is in total length of the allele. We added a sentence to the main text before this result to clarify this.

5. "genotypes obtained from ONT duplex and PacBio HiFi (88% allowing for 1bp off; Fig. 3a) with the latter showing higher concordance with the assembly (99% for PacBio HiFi vs 87% for ONT, allowing for 1bp off).": Earlier in the manuscript when comparing PacBio HiFi reads to the assembly, the authors mentioned that "LongTR showed […] 98.3% length concordance allowing for 1 bp differences." Can the authors clarify the accuracy of their tool?

We apologize for the confusion. The difference in numbers stems from slightly different TR loci being included in each of these analyses. The reported numbers for the ONT/PacBio comparison were derived from loci where LongTR calls were obtained from both the ONT and PacBio datasets, and where alleles could be extracted from the assembly. This analysis considered a total of 798,291 loci on autosomal chromosomes. On the other hand, for the TRGT/LongTR/assembly comparison, the analysis was confined to regions where calls were obtained from all three methods. This subset had 812,074 repeats. We have added the number of loci considered in each case to clarify.

6. In "Phasing information", How did authors determine the 20% cutoff for unphased reads?

This cutoff is somewhat arbitrary but was determined by manually inspecting reads in IGV for cases where incorporating phase information resulted in incorrect genotypes.

7. The figure legends for Fig 2.a. and Fig. 2.b seem to be swapped; 2a shows mendelian consistency, while 2b shows Concordance with genotype assembly.

Thanks for finding this issue. Note due to the journal requirement of a maximum of two figures for this format, we have moved one of the main figures to the supplementary material and so this is now Figure 1. We have now fixed this and reversed the order of the legend to match the figure.

8. Figure 3 needs to be replaced with higher resolution images.

This is now fixed (note this is now Figure 2).

Reviewer 2

Jam et al present LongTR, a method for genotyping short tandem repeats (STRs) and variable number tandem repeats (VNTRs) in long read data. The method is benchmarked against TRGT, a competing tool, and the performance of the method is demonstrated on both PacBio and ONT data. It is a timely work of interest to researchers in the field, and contains promising advances. I would draw the authors' attention to:

1. A number of STR and VNTR tools are cited, but not included in benchmarking. It would be beneficial to include a comparative evaluation of LongTR against all cited tools, specially STRaglr, given its integration into ONT's Epi2Me pipelines. This should be completed for publication of any bioinformatics tool in a field with multiple existing methods.

We benchmarked LongTR against Tandem-genotypes and Straglr and now report the results in the main text. We were unable to install and run TRiColor, and it seems that their GitHub repository is no longer maintained.

2. It should be noted that LongTR is benchmarked against TRGT v0.4.0. This is not the version of TRGT that ultimately is the subject of the cited TRGT paper (v0.5.0). A repeat of the benchmarking of LongTR against TRGT v0.5.0 is needed prior to publication. TRGT's current version is 0.8.0; significantly newer than that which LongTR is compared to here.

All benchmarking has been performed against TRGT v0.5.0. We mistakenly cited the older version which we used before this one was released. We have corrected the methods accordingly.

3. Please include information about how the software peformance was tested - computing environment, CPUs, total and type of memory used, and whether software was run in isolation (if the computing environment is shared, such as a HPC where other jobs' IO requirements may impact benchmarking). This is needed for reproducibility and clarity.

We have now added the HPC hardware specification in the methods section “Benchmarking against TRGT”.

Minor/tyopgraphical

1. The URL in the review copy abstract has an extra period at the end of the github URL, which causes it to fail.

Thanks for catching this, it is fixed now.

2. I found the order of presentation of LongTR performance difficult to follow in places. I would suggest presenting this as a benchmark against ground truth sequence first, then against competing software, rather than the reverse.

We have revised the text to present results in the suggested order, with the assembly comparison first followed by comparisons to competing software.

3. Are the reads that can be resolved with longer ONT reads but not shorter PacBio reads in known hard-to-map regions for long read sequencing, such as those previously identified by the GIAB Consortium (e.g.

https://urldefense.com/v3/__https://www.cell.com/cell-genomics/pdfExtended/S2666-979X(22)0

0058-1)?__;!!LLK065n_VXAQ!harfAJJatqI7EW-DhErFhTQGn1S0RpjkvTd3gxFsX2c-s85WgTBu

CdqG4-SuC-GGaJaq2B8A10Sl-OI6Wyg$

We checked the overlap with hard-to-map regions. Out of 2,573 repeats that were resolved by ONT and not PacBio, 938 were overlapping with one hard-to-map region, compared to 77,035 overlap across all 798,291 regions in comparison, indicating indeed that these repeats are indeed enriched in hard-to-map regions (Fisher exact test two-sided p-value of 1.75e-292). We have now noted this in the main text.

4. The release notes for TRGT v0.5.0 mention the incorporation of SNP information similar to the haplotag read phase information used by LongTR and TRiCoLOR. The statement on page 4 regarding the uniqueness of LongTR's approach among long read TR genotypers in using read phase information needs revision.

We have revised this paragraph to mention the read phase earlier on so we no longer imply the use of haplotag information is unique to LongTR.

5. LongTR needs to be packaged into a release on Github and a version number assigned prior to publication. Please release the version of the software described in the manuscript as version 1.0 per standard semantic versioning for released software.

We released LongTR v0.0.1 on GitHub.

**2^nd^ round**

**Reviewer 1**

The authors have satisfactorily addressed my previous comments. I have no further comments.

**Reviewer 2**

I would like to thank the authors for addressing my previous concerns.

The only remaining point that I'd raise is that LongTR should be released at v1.0, not v0.0.1; the Semantic Versioning standard states: "Major version zero (0.y.z) is for initial development. Anything MAY change at any time. The public API SHOULD NOT be considered stable." - https://semver.org/

Once this is corrected then I can wholeheartedly recommend publication.

**Authors’ response**

1. We edited “tandem repeat” in the title to “tandem repeats”

2. The reference publication for HG002 assembly v1.0.1 has been changed (reference

number 10).

3. In the sentence beginning “Of 814,319 repeats for…”, a metric for sequence

concordance between LongTR and assembly is reported, in addition to length

concordance. This is because the sequence preceding that mentions both sequence-

level and length-level comparisons are possible but previously only the length-based

metric was mentioned.

4. A reference publication for Nanopore Duplex data has now been added (reference

number 19).

5. In the section on comparison to HipSTR in results and discussion, we changed the

sentence from “For shorter repeats, concordance (by allowing 1bp off) decreases with increasing length of the repeat (Additional file 1: Fig. S7b).” to “Concordance (by

allowing 1bp off) decreases with increasing length of the repeat (Additional file 1: Fig.

S7b).” as the Fig S7b shows the trend for all repeats, not only the shorter repeats.

6. In the methods section for comparison to HipSTR, we removed the specifications of

running HipSTR on PacBio HiFi reads, as we only reported the results from running

HipSTR on Illumina short reads.

Based on Reviewer 2’s suggestion, we have updated the release version number of

LongTR to v1.0.
